# A weekly regimen of cisplatin, paclitaxel and topotecan with granulocyte-colony stimulating factor support for patients with extensive disease small cell lung cancer: a phase II study

**DOI:** 10.1054/bjoc.2001.1741

**Published:** 2001-05

**Authors:** G Frasci, G Nicolella, P Comella, I Carreca, G DeCataldis, D Muci, C Brunetti, M Natale, F Piantedosi, A Russo, S Palmeri, G Comella, N Panza

**Affiliations:** Southern Italy Cooperative Oncology Group (SICOG)-c/o National Tumor Institute of Naples, via M. Semmola 80131, Italy

**Keywords:** small cell lung cancer, weekly chemotherapy, paclitaxel, topotecan

## Abstract

The present study was aimed at defining the antitumour activity of the cisplatin-paclitaxel-topotecan (CPT) weekly administration with G-CSF support in chemo-naive SCLC patients with extensive disease (ED-SCLC). Chemonaive ED-SCLC patients received cisplatin 40 mg/m^2^, paclitaxel 85 mg/m^2^, and topotecan 2.25 mg/m^2^weekly, with G-CSF (5 μg/kg days 3–5) support, for a maximum of 12 weeks. 37 patients were treated, for a total of 348 cycles delivered. 8 complete responses (22%) and 22 partial responses (59%) were recorded, giving an 81% [95% CI = 65–92%] ORR. At a 13-month (range, 4–26) median follow-up, median progression-free and overall survival were 8 months and 12.5 months, with 1-year and 2-year projected survivals of 55% and 21%, respectively. No toxic deaths occurred. Grade 4 neutropenia and thrombocytopenia occurred in 6 and 3 patients, respectively. Only one case of neutropenic sepsis was recorded, while haemorrhagic thrombocytopenia was never observed. Diarrhoea, paraesthesias and fatigue were the main nonhaematologic toxicities being severe in 6, 2 and 10 patients, respectively. The weekly CPT combination with G-CSF support represents a well tolerated therapeutic approach in chemo-naive ED-SCLC patients. The activity rate seems at least similar to that achievable with the standard front-line approaches. © 2001 Cancer Research Campaign http://www.bjcancer.com


*British Journal of Cancer* (2001) **84**(9), 1166–1171
© 2001 Cancer Research Campaign
doi: 10.1054/ bjoc.2000.1741, available online at http://www.idealibrary.com on IDEAL®

http://www.bjcancer.com

### A weekly regimen of cisplatin, paclitaxel and topotecan with granulocyte-colony stimulating factor support for patients with extensive disease small cell lung cancer: a phase II study

**G Frasci, G Nicolella, P Comella, I Carreca, G DeCataldis, D Muci, C Brunetti, M Natale, F Piantedosi, A Russo, S Palmeri, G Comella and N Panza**

Southern Italy Cooperative Oncology Group (SICOG)-c/o National Tumor Institute of Naples, via M. Semmola 80131, Italy

**Summary** The present study was aimed at defining the antitumour activity of the cisplatin-paclitaxel-topotecan (CPT) weekly administration with G-CSF support in chemo-naive SCLC patients with extensive disease (ED-SCLC). Chemonaive ED-SCLC patients received cisplatin 40 mg/m$^2$, paclitaxel 85 mg/m$^2$, and topotecan 2.25 mg/m$^2$ weekly, with G-CSF (5 µg/kg days 3–5) support, for a maximum of 12 weeks. 37 patients were treated, for a total of 348 cycles delivered. 8 complete responses (22%) and 22 partial responses (59%) were recorded, giving an 81% [95% CI = 65–92%] ORR. At a 13-month (range, 4–26) median follow-up, median progression-free and overall survival were 8 months and 12.5 months, with 1-year and 2-year projected survivals of 55% and 21%, respectively. No toxic deaths occurred. Grade 4 neutropenia and thrombocytopenia occurred in 6 and 3 patients, respectively. Only one case of neutropenic sepsis was recorded, while haemorrhagic thrombocytopenia was never observed. Diarrhoea, paraesthesias and fatigue were the main nonhaematologic toxicities being severe in 6, 2 and 10 patients, respectively. The weekly CPT combination with G-CSF support represents a well tolerated therapeutic approach in chemo-naive ED-SCLC patients. The activity rate seems at least similar to that achievable with the standard front-line approaches. © 2001 Cancer Research Campaign http://www.bjcancer.com

**Keywords**: small cell lung cancer; weekly chemotherapy; paclitaxel; topotecan

Lung cancer still represents one of the most relevant health problems in Western countries, being the most common cause of cancer-related death. Small cell lung cancer (SCLC) accounts for 25% of all lung cancers (Landis et al, 1998). The majority of SCLC patients already have extensive disease at diagnosis.

In spite of more than two decades of intensive investigation with various chemotherapy combinations, the prognosis of these patients remains very poor, the median survival time still being shorter than one year, and the probability of remaining alive after 5 years being remote (Johnson et al, 1990).

Recently, several new agents have demonstrated activity against SCLC.

Topotecan is a semisynthetic hydrophilic analogue of camptothecin, an alkaloid derived from the oriental tree *Camptotheca acuminata*. It exerts its cytotoxic effects through the inhibition of the nuclear enzyme topoisomerase I (Chen and Liu, 1994). Single-agent topotecan, administered as a short-term infusion daily × 5 days every 3 weeks, has produced remarkable results in either chemo-naive or pretreated SCLC patients (Schiller et al, 1996; Ardizzoni et al, 1997). Although this schedule has been the most commonly used in phase II trials, other schedules like the 24-hour infusion weekly administration, the 21-day low-dose continuous infusion etc. have been testing in the last few years, giving interesting results (Slichenmeyer et al, 1993; Burris et al, 1994; Haas et al, 1994; Hochster et al, 1994).

Paclitaxel has also been tested as single agent in SCLC patients. In two phase II studies it produced an ORR of 34% and 41% in unpretreated patients with extensive disease (Kirshling et al, 1994; Ettinger et al, 1995). It has also been tested in combination with cisplatin (or carboplatin) and etoposide with promising preliminary results (Levitan et al, 1995; Kelly et al, 1996; Hainsworth et al, 1997; Glisson et al, 1999).

It has recently been reported that the weekly short-term (1- or 3-hour) administration of paclitaxel may result in a dramatic increase of the dose-density, at a price of a moderate toxicity (Fennelly et al, 1997; Akerley et al, 1998; Sikov et al, 1998).

The combination of topotecan and paclitaxel was tested in chemo-naive SCLC patients, showing a very promising ORR and median survival. However, haematologic toxicity proved to be relevant, and prolonged G-CSF support was required (Jett et al., 1997).

In view of the relevant myelotoxicity associated with both the topotecan-cisplatin (Rowinsky et al, 1997) and topotecan–paclitaxel combinations (O'Reilly et al, 1997) when the standard 5-day topotecan administration is used, we previously conducted a phase I study testing the simultaneous weekly administration of cisplatin, paclitaxel and topotecan (CPT). In the presence of G-CSF support from days 3 to 5 of each week, doses of cisplatin 40 mg/m$^2$, paclitaxel 85 mg/m$^2$ and topotecan 2.25 mg/m$^2$ were given weekly at a price of mild haematologic and nonhaematologic toxicity (Frasci et al, 1999).

*Received 12 September 2000*
*Revised 2 January 2001*
*Accepted 22 January 2001*

*Correspondence to:* G Frasci

In view of the promising therapeutic activity observed in the phase I study in both chemo-naive and pretreated SCLC patients, we have undertaken this phase II study aimed at better defining the efficacy of this new regimen as a front-line approach in patients with extensive-disease small cell lung cancer.

### METHODS

### Eligibility criteria

Candidates for this study were patients with histologically/cytologically proven SCLC who had not received prior chemotherapy or radiotherapy. Disease had to extend beyond the hemithorax of origin and regional lymph-nodes. Other eligibility criteria included clinically or radiologically measurable or evaluable disease, age between 18 and 75 years, Zubrod performance status of 0 to 2, life expectancy of at least 12 weeks, no previous or concurrent malignancy, except for inactive non-melanoma skin cancer, and in situ carcinoma of the cervix.

Adequate bone marrow (absolute neutrophil count $>2 \times 10^9$/l, platelet count $>100 \times 10^9$/l, and haemoglobin level >10 g $l^{-1}$), liver (bilirubin level <2 times the upper normal limit, AST and/or ALT <3 times the upper normal limit, prothrombin time <1.5 times control), and renal function (creatinine clearance >60 ml $min^{-1}$) were also required. The presence of severe cardiac arrhythmia or heart failure, second- or third-degree heart block or acute myocardial infarction within 4 months prior to study entry were considered as exclusion criteria. CNS metastases were not considered an exclusion criterion, provided that a good symptomatic control could be achieved with steroids. All patients gave their witnessed written informed consent, and the protocol was approved by the Ethical Committee for the Biologic Research of the National Tumor Institute of Naples.

### Pretreatment workup

Pretreatment evaluation included a complete history and physical examination, determination of haematology, chemistry, and tumour marker levels (CEA, TPA, NSE), ECG, chest X-ray, brain, chest and abdomen CT, radionuclide scan of bone and bronchoscopy. Additional radiologic examinations were also performed as necessary to document the extent of disease.

### Treatment

All eligible patients received paclitaxel 85 mg/m$^2$ (as a 1-hour infusion) followed by cisplatin 40 mg/m$^2$ and topotecan 2.25 mg/m$^2$ as a 30-min i.v. infusion, weekly. Prophylaxis for hypersensitivity reactions consisted of dexamethasone 8 mg i.v. 1 hour and promethazine 50 mg i.v. plus ranitidine 50 mg i.v. 30 minutes before paclitaxel administration. G-CSF was given at the dose of 5 μg/kg$^{-1}$ day$^{-1}$ days 3–5 of each week. A minimum of 6 treatment administrations were delivered. Patients showing a clinical complete or partial response received additional 6 administrations. In absence of a major tumour regression patients were shifted to a cyclophosphamide-doxorubicin-etoposide treatment. This regimen was also used for patients relapsing after initial response.

Patients with brain metastasis who developed progressive disease at any time, or did not have a major response in the brain after 6 administrations, received whole brain radiotherapy. In patients who had a major response in the brain after 6 administrations, whole brain radiotherapy was deferred until after the 12th administration.

### Adjustments according to toxicity

Chemotherapy was given at full doses if neutrophil count was ≥1500/mm$^2$, and platelet count ≥100 000/mm$^2$. For neutrophil count >1000/mm$^2$, or platelet count >75 000/mm$^2$, it was delivered at 50% of the planned dose. For neutrophil count <1000/mm$^2$, or platelet count <75 000/mm$^2$ chemotherapy was always omitted on that day. In presence of grade 4 neutropenia lasting more than 7 days, febrile neutropenia (>38.5˚C with ANC <500), grade 4 thrombocytopenia (lasting longer than 4 days), grade 4 anaemia, grade 3–4 nonhaematologic toxicity (except for nausea/vomiting and alopecia) the weekly chemotherapy dose was reduced to 75% in the subsequent administrations. Dose reduction was not performed for shorter than 4 days severe myelosuppression.

### Toxicity and response assessment

Toxicities and response were graded according to the WHO criteria (Miller et al, 1981).

Haematologic toxicity was assessed by performing weekly blood cell count and differential. In cases of grade 4 haematological toxicity occurrence these tests were performed every other day.

For each patient, the worst toxicity encountered during the treatment was reported.

The clinical restaging was performed after 6- and 12-weekly administrations. It consisted of physical examination, routine laboratory tests, serum tumour markers, and repetition of all initially abnormal diagnostic procedures. For bone metastases, the standard WHO response criteria were applied: CR was considered as the complete disappearance of all lesions on X-ray or scan for at least 4 weeks; PR was defined as a greater than 50% reduction in the number of the areas of uptake from the pretreatment radionuclide scan, or partial decrease (>50%) in size of lytic lesions, recalcification of lytic lesions, or decreased density of blastic lesions for at least 4 weeks.

All eligible patients were included in the response and survival analysis, on an ‘intent to treat’ basis. Duration of complete response was calculated from the date of the first documentation of CR until date of disease progression; duration of partial response, and time to treatment failure were both calculated from the day of initial treatment until PD was firstly noted. Overall survival (OS) was calculated from the beginning of treatment to death by any cause.

For time-to-event data, the cumulative proportion of patients who had not yet experienced the event was plotted as function of time by means of the Kaplan–Meier product-limit approach (Kaplan and Meier, 1958).

### Study design

Since we believe that the achievement of a complete response often translates in a prolongation of survival in SCLC patients, we have chosen the cCR rate as the main end-point of this study.

We aimed at obtaining a 30% cCR rate with this new combination, and set a cCR rate of 10% as the lowest level of interest. According to the Simon two-stage minimax design (Simon, 1989), at least 7 complete responses were required among 33 patients to

© 2001 Cancer Research Campaign 

consider this combination worth of further evaluation (alpha = 0.05; 1-beta = 90%). A first analysis was planned after 22 patients had been enrolled. The accrual would have been stopped if fewer than 3 complete responses had been observed.

### RESULTS

### Demographics

Between August 1997 and July 1999 3 patients entered the study.

Table 1 outlines the main patient characteristics. 29 patients were male. Median age was 51 (range, 38–76) years, and 14 patients (38%) had a Zubrod performance status of 2. Clinically asymptomatic brain involvement was present in 10 (27%) patients, and 23 (62%) patients had increased LDH serum levels. 22 (59%) patients had 3 or more sites of involvement.

### Response

All the 37 enrolled patients were evaluated for response on an 'intent to treat' basis.

33 patients completed at least 6 administrations and were assessed for response. 4 patients did not complete the first 6 administrations due to disease progression (2 cases), protracted thrombocytopenia (1 case), or refusal (1 case). All of them were considered as failures.

An overall, 8 complete (22%) and 22 partial responses (59%) were recorded, for a 81% [95% CI = 65–92%] overall response rate (Table 2). All the 8 complete responses occurred in the first 33 enrolled patients. 6 complete responses were achieved after 6 administrations. In another 2 patients residual tumour persisted in lung and liver after 6 cycles, but disappeared by the 12th administration. Liver was the main site of disease involvement in 5/8 complete responders, the tumour being localized in bone (2) and adrenal (1) in the remaining 3 cases.

26 out of 30 responder patients received the planned 12 administrations of chemotherapy. In 3 patients chemotherapy was suspended because of the occurrence of severe fatigue (2), or peripheral neuropathy (1), while another patient refused treatment after 8 administrations.

7 of the 10 patients with brain metastasis achieved a major response after 6 cycles. In 5 of these cases a major tumour regression was also observed in the brain, but it was never complete.

Of the 14 patients with poor performance status, overall 6 major responses (43%) were recorded, only one being complete.

**Table 2** Responses (37 assessable patients)

| | No. of patients |
|---|---|
| Complete responses (%) | 8 (22) |
| Partial responses (%) | 22 (59) |
| Stable disease (%) | 2 (5.5) |
| Progressions (%) or treatment failures | 5 (13.5) |
| Overall responses (%) | 30 (81) |

**Table 1** Patient characteristics

| Characteristic | | No. of patients |
|---|---|---|
| Age, years | | |
| Median | 51 | |
| Range | 38–76 | |
| Sex | | |
| Male | | 29 |
| Female | | 8 |
| ECOG performance status | | |
| 0 | | 6 |
| 1 | | 17 |
| 2 | | 14 |
| Site of metastasis | | |
| Contralateral lung | | 21 |
| Extrathoracic lymph-nodes | | 4 |
| Liver | | 26 |
| Brain | | 10 |
| Bone | | 24 |
| Adrenal | | 8 |
| No. of disease sites | | |
| 1–2 | | 15 |
| ≥3 | | 22 |
| LDH serum levels | | |
| Normal | | 14 |
| Increased | | 23 |
| Total | | 37 |

### Survival

At the time of the present analysis 5 patients were still progression-free and 11 were still alive, with a median follow-up time of 13 (range, 4–26) months in the survivors. Actuarial estimation of median failure-free and overall survival for the entire population was 8 months [95% CI = 6–9] and 12.5 months [95% CI = 8–14], the 1-and 2-year survival rates were 55% and 21%, respectively (Figures 1 and 2). The one-year survival probability was 68%, 61% and 29% in complete responders, partial responders, and nonresponders, respectively. Among the 8 patients still alive at a >18-month follow-up, 4 had achieved a complete response, and 3 a partial response, while the last one had failed to respond either to first-line or second-line treatment. The 14 patients with Zubrod performance status 2 experienced a substantially shorter median survival time (8 months) than those with better performance status (14 months). A worse survival outcome was also seen in the 23 patients with increased LDH serum levels at diagnosis. Indeed, median survival was 7.5 months in this group, as compared to 14 months in the others. The median survival time was slightly shorter in the 10 patients with brain metastasis (10 months) as compared to the others (13 months).

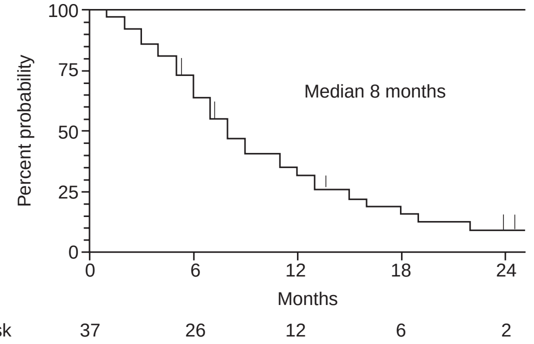

**Figure 1** Progression-free survival. Total patients = 37; progression-free = 5

 © 2001 Cancer Research Campaign

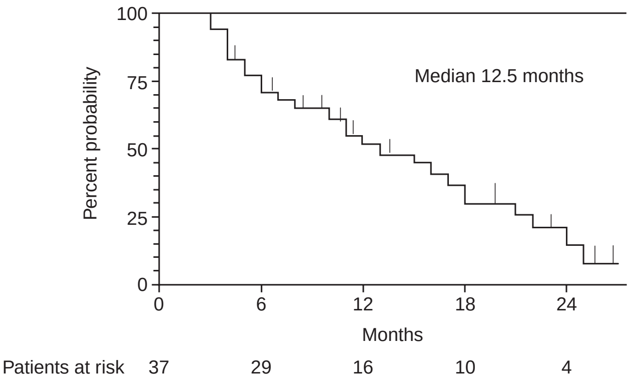

**Figure 2** Overall survival. Total patients = 37; alive = 11

### Toxicity

Table 3 summarizes the myelotoxicity data on the overall 348 administrations.

No toxic deaths were observed. Only one case of neutropenic fever was recorded, after 9 chemotherapy administrations, in a patient with massive liver and bone involvement. Overall, grade 3 and 4 neutropenia occurred in 10 (27%) and 6 (16%) patients, respectively. Thrombocytopenia was less relevant, being of grade 3 in 7 patients (19%), while grade 4 never occurred. However, in one patient (with a concomitant chronic hepatitis) a persistent grade 3 thrombocytopenia occurred after 2 administrations, which caused the definitive suspension of chemotherapy. Anaemia was quite frequent especially in patients who received more than 6 administrations. Haemoglobin levels fell below 11 g $l^{-1}$ in all patients, and in 7 patients (19%) a packed red blood cell transfusion was required.

Alopecia was very frequent, occurring in 33 of the 37 enrolled patients. Other nonhaematologic toxicities were generally mild/moderate. Severe hypersensitivity reactions to paclitaxel were not observed. Gastrointestinal toxicity was moderate in the majority of patients and never led to definitive treatment discontinuation. Grade 3 vomiting and diarrhoea occurred in 4 patients (11%) and 7 patients (19%), respectively.

A 25% reduction of all 3 drug doses was performed in patients showing severe diarrhoea and this prevented a further occurrence of this side effect.

Severe constipation was observed in 6 patients, and an additional 3 patients had moderate transient elevation of hepatic enzymes. Severe fatigue occurred in 10 patients, and in 2 cases it led to the definitive suspension of the treatment (both patients were responsive after the first 6 chemotherapy cycles). Neurologic toxicity was not a problem in the majority of patients. Mild peripheral paraesthesia occurred in 12 patients (32%) and in an additional 4 patients (11%) a grade 2 neurotoxicity was observed. Only 2 patients showed grade 3 peripheral neuropathy: in one case it occurred after 8 cycles, causing the discontinuation of chemotherapy, the other patient showed a worsening of peripheral neuropathy after the completion of treatment. In both cases neurotoxicity persisted for several months.

Musculoskeletal symptoms like transient arthralgia and myalgia were quite frequent, occurring in an overall 19 patients (51%) but they were never severe, and responded well to anti-inflammatory drugs.

Nephrotoxicity was almost non-existent: only 4 cycles were associated with a mild and transient elevation of creatinine serum level.

Mucositis was not uncommon, but it was never severe. One or more episodes of rhinorrhagia occurred in 13 patients.

**Table 3** Worst acute toxicity (37 patients)

| Toxicity | WHO grade | | | |
|---|---|---|---|---|
| | 1 | 2 | 3 | 4 |
| Haemoglobin | 13 | 17 | 6 | 1 |
| ANC | 6 | 15 | 10 | 6 |
| Platelets | 15 | 12 | 7 | 0 |
| Vomiting | 20 | 11 | 4 | 0 |
| Diarrhoea | 8 | 9 | 6 | 0 |
| Neuropathy | 12 | 4 | 2 | 0 |
| Mucositis | 9 | 5 | 0 | 0 |
| Fatigue | 15 | 9 | 10 | 0 |
| Alopecia | 11 | 15 | 7 | 0 |

### DISCUSSION

The haematologic data of the present study confirms the findings of our previous phase I trial testing this new weekly combination (Frasci et al, 1999). Indeed, the occurrence of severe neutropenia or thrombocytopenia was negligible with this combination, while anaemia, although more frequent, actually cannot be considered as a life-threatening toxicity. Severe nonhaematologic toxicity was also uncommon. Gastrointestinal and neurologic side effects were frequent, but rarely compromised the continuation of the treatment.

In view of the high chemosensitivity of SCLC, we did not consider the rate of objective responses as a reliable parameter to evaluate the capability of this new regimen to improve the prognosis of SCLC patients. Indeed, ORRs higher than 80% have also been reported in the past with the use of standard combinations not including new agents (Johnson et al, 1990).

In order to ascertain whether our new combination could be a real step ahead in the treatment of SCLC patients, we set a quite restrictive criterion to pursue in future phase III trials. Indeed, we set a 30% complete response rate as the target activity rate, and considered a complete response rate with a 95% confidence interval which fell below 10% as uninteresting.

A high proportion of patients has shown a major regression of the tumour in the present trial, and the number of complete responses observed (8), although not very impressive, exceeded the minimum required by the study design. Moreover, it deserves to be remarked that all patients showed disseminated disease at beginning of chemotherapy.

The short duration of the treatment could have had a role in determining this unimpressive complete response rate.

The presence of poor prognostic features at beginning of treatment like poor performance status, brain metastases, and elevated LDH serum levels could also have had a substantial role in underestimating the CR rate. Indeed, about 30% of patients treated by us had brain metastasis, and none obtained a complete regression of the tumour in the brain. The relevant number of patients with poor performance status could also have contributed to reducing the ORR and the CR rate of the whole population, since they were 43% and 7% in this group.

The survival outcome of our patients deserves some additional comments. Of course the absence of a randomization makes it

© 2001 Cancer Research Campaign difficult to draw definite conclusions on this issue; however, we think that the median survival time observed in our study population is interesting. Indeed, if we look at the previous experiences conducted either with ‘old’ or with ‘new’ combinations, the achievement of a longer than 1-year median survival time was very uncommon in extensive-disease patients. Moreover, as previously remarked, our study population was not at all a ‘good-prognosis’ one. In fact, the survival outcome looks much better in our study if patients with poor performance status or with elevated LDH serum levels are excluded from the analysis, in these cases the median survival times are 14 months and 15 months, respectively.

Although it is impossible, on the basis of our results, to conclude that the addition to cisplatin of paclitaxel and topotecan can substantially modify the life expectancy of ED-SCLC patients, it must be remarked that also in other trials which tested the topotecan-paclitaxel regimen a good median survival was seen, even when an unimpressive ORR and CR rate were obtained (Jacobs et al, 1999; Lynch et al, 1999; Tweedy et al, 1999). However, the results of the recent CALGB 9430 phase II trial testing the paclitaxel–topotecan combination do not suggest an advantage to this regimen compared to standard etoposide and platinum (Lynch et al, 2000).

The use of weekly rather than daily times 5 every 3 weeks topotecan was a peculiarity of the present study. It is interesting to remark that the cumulative 3-week dose of topotecan in our study (6.75 mg/m$^2$) was much higher than those reported in other trials testing the topotecan in two-or three-drug regimens (O’Reilly et al, 1997; Rowinsky et al, 1997; Dunphy et al, 1998; Ten Bokkel Huinink et al, 1998; Raefsky et al, 1999). Other phase II trials testing the cisplatin–paclitaxel–topotecan regimen in SCLC patients have not been conducted. However, in a phase 1 study from the Netherlands the cisplatin–paclitaxel–topotecan triple combination was also tested, by using the standard every 3-week schedule for all 3 drugs. In presence of G-CSF support doses of cisplatin and paclitaxel of 75 mg/m$^2$ and 110 mg/m$^2$ (24-hour) were given, and the dose of TPT did not exceed 0.4 mg/m$^2$ d 1–5 every 3 weeks (Ten Bokkel Huinink et al, 1998). Preliminary phase I experiences in refractory solid tumours with the carboplatin–paclitaxel–topotecan combination have also been reported. A standard daily times 3 or 5 every 3 weeks schedule had always been performed. The recommended dose of TPT was 0.5 mg/m$^2$/d $\times$ 5 days, or 0.75 mg/m$^2$/d $\times$ 3 days, in both cases giving a cumulative over 3-week dose less than half of that delivered by us (Raefsky et al, 1999; Dunphy et al, 1998). Preliminary phase II results of this regimen in small cell lung cancer have also been recently reported. An 88% overall response rate was obtained in patients with extensive disease, although patients with poor performance status tolerated treatment poorly (Gray et al, 2000).

The very good tolerance of weekly topotecan given in a short-term infusion as single agent has been recently confirmed by the preliminary data of a phase I study, although activity data were not reported (Clark et al, 1999). Therefore, further confirmations of the efficacy of this schedule are required.

In conclusion, the results of the present phase II study suggest that 2 new drugs like paclitaxel and topotecan can be safely combined at full doses with cisplatin, by using a weekly schedule with G-CSF support. This combination results in both a good ORR and promising median survival time. Only a large phase III trial will establish whether this dose-dense chemotherapy approach can produce a substantial prognostic improvement in extensive-stage SCLC patients.

### REFERENCES

Akerley W, Glantz M, Choy H, Rege V, Sambandam S, Joseph P, Yee L, Rodrigues B, Wingate P and Leone L (1998) Phase I trial of weekly paclitaxel in advanced lung cancer. *J Clin Oncol* **16**: 153–158

Ardizzoni A, Hansen H, Dombernowsky P, Gamucci T, Kaplan S, Postmus P, Giaccone G, Schaefer B, Wanders J and Verweij J (1997) Phase II study of topotecan in pretreated small cell lung cancer. *J Clin Oncol* **15**: 2090–2096

Burris HA, Awada A, Kuhn JG (1994) Phase I and pharmacokinetic studies of topotecan administered as 72 or 120 hr continuos infusion. *Anticancer Drugs* **5**: 394–402

Chen AY and Liu LF (1994) DNA topoisomerases: essential enzymes and lethal targets. *Ann Rev Pharmacol Toxicol* **34**: 191–218

Clark RS, Fracasso PM and Picus J (1999) A phase I study of weekly topotecan as a bolus infusion. *Proc Am Soc Clin Oncol* **18**: 207a (abstract 793)

Dunphy F, Dunleavy T, Turcotte C, Petruska P and Pincus S (1998) Phase I study of topotecan plus carboplatin-paclitaxel in solid tumors. *Proc Am Soc Clin Oncol* **17**: 240a (abstract 919).

Ettinger DS, Finkelstein DM, Sarma RP. et al (1995) Phase II study of paclitaxel in patients with extensive-disease small-cell lung cancer: An Eastern Cooperative Oncology Group study. *J Clin Oncol* **13**: 1430–1435

Fennelly D, Aghajanian C, Shapiro F, O’Flaherty C, McKenzie M, O’Connor C, Tong W, Norton L and Spriggs D (1997) Phase I and pharmacologic study of paclitaxel administered weekly in patients with relapsed ovarian cancer. *J Clin Oncol* **15**: 187–192

Frasci G, Panza N, Comella P, Cartenì G, Guida T, Nicolella GP, Natale M, Lombardi R, Apicella A, Pacilio C, Gravina A, Lapenta L and Comella G (1999) Cisplatin-topotecan-paclitaxel weekly administration with G-CSF support for ovarian and small-cell lung cancer patients. A dose-finding study. *Ann Oncol* **10**: 355–358.

Glisson BS, Kurie JM, Perez-Soler R, Fox NJ, Murphy WK, Fossella FV, Les JS, Ross MB, Nyberg DA, Pisters KMW, Shin DM and Hong WK (1999) Cisplatin, etoposide, and paclitaxel in the treatment of patients with extensive small-cell lung carcinoma. *J Clin Oncol* **8**: 2309–2315

Gray JR, Hainsworth JD, Burris HA, Morrissey LH, Gualtieri R, Joseph G, Steis R and Greco FA (2000) Paclitaxel, Carboplatin, and Topotecan in the treatment of Small Cell Lung Cancer: A phase II trial of the Minnie Pearl Cancer Research Network. *Proc Am Soc Clin Oncol* **19**: 494a. (abstract 1931)

Haas NB, LaCreta FP, Walczak J, Hudes GR, Brennan JM, Ozols RF and O’Dwyer PJ (1994) Phase I/pharmacokinetic study of topotecan by 24-hour continuous infusion weekly. *Cancer Res* **54**: 1220–1226

Hainsworth JD, Gray JR, Stroup SL, Kalman LA, Patten JE, Hopkins LG, Thomas M and Greco FA (1997) Paclitaxel, carboplatin, and extended-schedule etoposide in the treatment of small-cell lung cancer: comparison of sequential phase II trials using different dose-intensities. *J Clin Oncol* **15**: 3464–3470

Hochster H, Liebes L, Speyer J, Sorich J, Taubes B, Oratz R, Werntz J, Chachoua A, Raphael B and Vinci RZ (1994) Phase I trial of low dose continuous topotecan infusion in patients with cancer: an active and well tolerated regimen. *J Clin Oncol* **12**: 553–559

Jacobs SA, Jett JR, Belani CP, Long JS, Day RD, Kim HD, Levitt ML and Wooley PV (1999) Topotecan and paclitaxel, an active couplet in untreated extensive disease small-cell lung cancer. *Proc Am Soc Clin Oncol* **18**: 470a. (abstract 1814)

Jett JR, Day R, Levitt M, Woolley G and Jacobs S (1997) Topotecan and paclitaxel in extensive stage small cell lung cancer patients without prior therapy. *Lung Cancer* **18** (suppl. 1): 13. (abstract 38)

Johnson BE, Grayson J, Makuch RW, Linnoila RI, Anderson MJ, Cohen MH, Glatstein E, Minna JD and Ihde DC (1990) Ten-year survival of patients with small-cell lung cancer treated with combination chemotherapy with or without irradiation. *J Clin Oncol* **8**: 396–401

Kaplan ES and Meier P (1958) Non parametric estimation for incomplete observations. *J Am Stat Assoc* **53**: 557–580

Kelly K, Wood ME and Bunn PA (1996) A phase I study of cisplatin etoposide and paclitaxel in extensive stage small-cell lung cancer. *Proc Am Soc Clin Oncol* **15**: 400. (abstract 1214)

Kirshling LJ, Jung SH and Jett JR (1994) A phase II trial of taxol and G-CSF in previously untreated patients with extensive stage small-cell lung cancer. *Proc Am Soc Clin Oncol* **13**: 326. (abstract 1076)

Landis SH, Murray T, Bolden S and Wingo PA (1998) Cancer statistics, 1998. *CA Cancer J Clin* **48**: 6–29

Levitan N, McKenney J, Tahsildar H and Ettinger D (1995) Results of a phase I dose escalation trial of paclitaxel, etoposide and cisplatin followed by filgrastim in the treatment of patients with extensive stage small cell lung cancer. *Proc Am Soc Clin Oncol* **14**: 379. (abstract 1177)

 *© 2001 Cancer Research Campaign*

Lynch TJ, Herndon J, Lilenbaum RC, Lyss A, Miller AA and Green MG (1999) Toxicity of paclitaxel and topotecan in patients with previously untreated extensive small-cell lung cancer. *Proc Am Soc Clin Oncol* **18**: 515a. (abstract 1987)

Lynch TJ, Herndon JE, Lyss AP, Mauer A, Watson D, Miller A, Lilenbaum R and Green MR (2000) Paclitaxel + topotecan + G-CSF for previously untreated extensive Small Cell Lung Cancer: preliminary analysis of Cancer and Leukemia Group B (CALGB) 9430. *Proc Am Soc Clin Oncol* **19**: 491a. (abstract 1922).

Miller AB, Hoogstraten B, Staquet M and Winkler A (1981) Reporting results of cancer treatment. *Cancer* **47**: 207–214

O' Reilly S, Fleming GF, Baker SD, Walczak JR, McGuire 3rd WP, Schilder RJ, Alvarez RD, Armstrong DK, Horowitz IR, Ozols RF and Rowinsky EK (1997) Phase I trial and pharmacologic trial of sequences of paclitaxel and topotecan in previously treated ovarian epithelial malignancies. *J Clin Oncol* **15**: 177–186

Raefsky EL, Hainsworth JD, Burris HA, Hopkins LG and Greco FA (1999) Phase I trial of topotecan, paclitaxel and carboplatin in patients with advanced refractory malignancies. *Cancer* **85**: 1179–1186

Rowinsky EK, Kaufman SH, Baker SD, Grochow LB, Chen TL, Peereboom D, Bowling MK, Sartorius SE, Ettinger DS, Forastiere AA and Donehower RC (1997) Sequences of topotecan and cisplatin phase I pharmacologic and in vitro studies to examine sequence dependence. *J Clin Oncol* **15**: 3074–3084

Schiller JH, Kim K, Hutson P, De Vore R, Glick J, Stewart J and Johnson D (1996) Phase II study of topotecan in extensive stage Small Cell carcinoma of the lung: an Eastern Cooperative Oncology Group Trial. *J Clin Oncol* **14**: 2345–2352

Sikov W, Akerley W, Strenger R and Cummings F (1998) Weekly high-dose paclitaxel demonstrates significant activity in advanced breast cancer. *Proc Am Soc Clin Oncol* **17**: 112a. (abstract 432)

Simon R (1989) Optimal two-stage designs for phase II clinical trials. *Control Clin Trials* **10**: 1–10

Slichenmyer WJ, Rowinsky EK, Donehouer RC and Kaufmann SH (1993) The current status of camptothecin analogoues as antitumor agents. *J Natl Cancer Inst* **85**: 271–291

ten Bokkel Huinink WW, Richel DJ, Herben VMM Panday VRN, Schellens JHM, van de Vange N, Beusenberg F, Hearn S, Beijnen JH and Rodenhuis S (1998) Feasibility study of the combination of cisplatin, paclitaxel and topotecan in ovarian cancer patients. *Proc Am Soc Clin Oncol* **17**: 351a. (abstract 1353)

Tweedy CR, Andrews DF and Ball T (1999) Topotecan and paclitaxel in extensive stage small-cell lung cancer as initial therapy. *Proc Am Soc Clin Oncol* **18**: 525a. (abstract 2025)

*© 2001 Cancer Research Campaign*
